# Plant extracts as alternatives for chemical disinfectants in veterinary settings in seven sub-Saharan African countries

**DOI:** 10.3389/fvets.2026.1895590

**Published:** 2026-09-30

**Authors:** Abass Elhadi, Alaa Ibrahim, Enass M. Abdalla, Julius B. Okuni, Victoria O. Adetunji, Akinlabi Ogunleye, George Ademowo, Yimtubezinash Woldeamanuel, Yakhya Dieye, Michael Frímpong, Sheila Makiala-Mandanda, Annemarie Kaesbohrer, Damalie Nakanjako, Ahmed Abd El Wahed, Uwe Truyen, Kamal H. Eltom

**Affiliations:** 1 Department of Surgery and Anaesthesia, Faculty of Veterinary Medicine, University of Khartoum, Khartoum North, Sudan; 2 Department of Animal Health and Safety of Animal Products, Institute for Studies and Promotion of Animal Exports, University of Khartoum, Khartoum North, Sudan; 3 Institute of Animal Hygiene and Veterinary Public Health, Leipzig University, Leipzig, Germany; 4 College of Veterinary Medicine, Animal Resources and Biosecurity, Makerere University, Kampala, Uganda; 5 Department of Veterinary Public Health and Preventive Medicine, University of Ibadan, Ibadan, Nigeria; 6 Department of Veterinary Microbiology, University of Ibadan, Ibadan, Nigeria; 7 Institute for Advanced Medical Research and Training College of Medicine, University of Ibadan, Ibadan, Nigeria; 8 CDT-Africa, College of Health Sciences, Addis Ababa University, Addis Ababa, Ethiopia; 9 Department of Microbiology, Immunology, and Parasitology, School of Biomedicine and Laboratory Sciences, College of Health Sciences, Addis Ababa University, Addis Ababa, Ethiopia; 10 Department of Microbiology, Institute Pasteur de Dakar, Dakar, Senegal; 11 Kumasi Centre for Collaborative Research in Tropical Medicine, Kwame Nkrumah University of Science and Technology, Kumasi, Ghana; 12 Institut National de Recherche Biomédicale, Kinshasa, Democratic Republic of Congo; 13 Cliniques Universitaires de Kinshasa, University of Kinshasa, Kinshasa, Democratic Republic of Congo; 14 German Federal Institute for Risk Assessment (BfR), Berlin, Germany; 15 Department of Medicine, College of Health Sciences, Makerere University, Kampala, Uganda

**Keywords:** *Acacia*/*Vachellia nilotica*, *Aloe vera*, *Citrus ×aurantiifolia*, *Hibiscus sabdariffa*, *Moringa oleifera*

## Abstract

**Background:**

Extracts of many medicinal plants possess antimicrobial activities and have been suggested in many studies as alternative antimicrobial agents to overcome the increasing antimicrobial-resistant bacteria worldwide. But the potential use of these medicinal plants in disinfection received little or no attention, especially in sub-Saharan Africa.

**Aim of the study:**

This review aimed at identifying from available literature candidate medicinal plants for use as alternatives for chemical disinfectants in resource-limited veterinary settings in seven African countries (Democratic Republic of Congo, Ethiopia, Ghana, Nigeria, Senegal, the Sudan and Uganda).

**Materials and methods:**

Online search in PubMed was conducted for literature from Africa on medicinal plants with reported antimicrobial activity. Then, five medicinal plants were selected based on documented antimicrobial activity of the aqueous extract, the plant part used for extraction, presence in all countries of the study, affordability and availability of sufficient online literature.

**Results:**

The selected plant species were *Vachellia nilotica* (L.)*, Aloe vera* (L.), *Citrus ×aurantiifolia*, *Hibiscus sabdariffa* (L.) and *Moringa oleifera* (Lam). Available online literature on these medicinal plants shows activity of their crude aqueous extracts against many bacteria, fungi and viruses, but provides few information on the minimum inhibitory concertation (MIC) of the extracts.

**Conclusion:**

The aqueous extracts of these medicinal plants can be prepared by simple techniques without sophisticated equipment. The long use of these medicinal plants in food or in ethnomedicine support their use as safe, accessible, sustainable and low-cost disinfectants in settings where chemical disinfectants are scarce, expensive, or supply-chain dependent. It should be emphasized that application conditions, concentrations and preparation methods require standardization and evaluation before practical use in disinfection.

## 1 Introduction

The Coronavirus Disease of 2019 (COVID-19) pandemic exposed major global vulnerabilities in the production, availability, and distribution of essential health and hygiene commodities, with supply-chain disruptions affecting access in many regions (1, 2). Dependence on imported health products and existing structural constraints made consistent access more difficult in low- and middle-income countries (3), especially African countries, in which the impact in their health systems was substantial (2). For example, a study in Tanzania on the preparedness of health facilities providing HIV-care during COVID-19 pandemic revealed low availability of alcohol-based hand rub and disinfectants, among other health supplies needs, especially in rural areas (4). Recurrence of similar pandemics or even continental confined disease outbreaks, such as Ebola hemorrhagic fever disease, is unavoidable. As disinfection is an essential measure in the prevention and control of infectious diseases, effective and locally available and affordable disinfectants, would be an important component of the preparedness for such disease outbreak situations.

Furthermore, the extensive use of chemical disinfectants and antiseptics during the COVID-19 pandemic, with their potential toxicity and environmental contamination risks reported in some countries, has renewed interest in identifying alternative green/eco-friendly disinfectants from natural sources, including medicinal plants (5–7). Such plant-derived disinfectants are sustainable and affordable, especially in regions, such as sub-Saharan Africa, where access to conventional disinfectants or medical supplies is often limited.

Medicinal plants have long been used in almost all over the world to manage infectious and non-infectious diseases (8). For example, Muhakr et al. (9) reported the ethnomedicinal use of *Boswellia papyrifera* resins, *Nigella sativa* seeds, *Vachellia* (*Acacia*) *nilotica* pods, and *Syzygium aromaticum* cloves for treating coronavirus infection in North Darfur, the Sudan, while Abubakar et al. (10) reported the frequent use of *Moringa oleifera, Elaeis guineensis*, *Acacia nilotica* by majority (70–80%) of the herbal medicine practitioners for the treatment of viral infections in Nigeria. The established ethnomedicinal use of these medicinal plants in the treatment of such infections indicates their antimicrobial potential. This antimicrobial potential of medicinal plants attracted the interest of researchers after discovery of the antibiotics in the third and fourth decades of the last century, as documented in early studies. For instance, the antimycobacterial effect of garlic (*Allium sativum*) extract was proposed and investigated by Rao et al. (11) on the grounds that it was known in the indigenous Unani and Ayurvedic systems of medicine for the treatment of tuberculosis. In the following decades, numerous studies documented the antimicrobial properties of several medicinal plants and identified bioactive phytochemical compounds therein. Subsequent research sought to identify the specific molecular targets and effects of individual phytochemical constituents. However, the constituents of medicinal plant extracts are often reported only as broad chemical classes, such as phenolics, tannins, alkaloids, sulfur-containing compounds, coumarins, etc.; each group comprises numerous individual compounds, only some of which have been isolated and identified. Accordingly, the antimicrobial mechanisms attributed to these chemical classes are usually described only in broad terms (12) and are inferred from the activity of some individual constituents of these groups (13–15). As medicinal plant extracts contain a mixture of several bioactive constituents possessing antimicrobial activity, the overall antimicrobial activity of a crude medicinal plant extract may reflect synergistic, additive, or antagonistic interactions among its constituents; moreover, some active constituents may be masked by others present in the same mixture. Therefore, the behavior of a complex extract cannot be explained by only a few known compounds and, accordingly, a defined mode of action cannot be assigned to crude plant extracts (16). In this respect, crude plant extracts with antimicrobial activity might resemble the biocides (disinfectants/ antiseptics), in that, “unlike chemotherapeutic antibiotics, biocides at their in-use concentration exert bactericidal activity by affecting multiple targets on the bacterial cell,” as Millard and Jascoe stated (17).

Ethnobotanical surveys documented the preparation of medicinal plant remedies from leaves, bark, roots, flowers, fruits and seeds using simple methods such as decoctions and infusions that require only water, heat, or basic household tools. These methods remain widely practiced in rural and peri-urban settings in Africa and are used to treat many gastrointestinal, respiratory, and fever-related diseases (9, 10) caused by pathogenic bacteria, viruses, fungi and protozoa. Therefore, the present review aimed at identifying from available online literature medicinal plant aqueous extracts with reported antimicrobial activities to explore their potential use as alternative antiseptics and disinfectants in low resource settings in Sub-Saharan Africa.

## 2 Materials and methods

Online search was made in PubMed for articles on medicinal plants from Africa with antimicrobial activity. The search was defined for the period from 1/1/2021 to 31/10/2025 and the advanced search function was used the key words ((((Medicinal) AND (Plant)) AND (Extract)) AND (Antimicrobial)) AND (Africa). The search resulted on 466 articles, from which 149 were excluded for one of the following reasons (1) not written in English language, (2) not from Africa (3) not about an indigenous African plant, (4) only on synthesis of nanoparticle using plant extracts, (5) no full text is available, and (6) the antimicrobial activity was not mentioned. A list of the identified medicinal plants was made from which five medicinal plants were selected based on: (1) reported antimicrobial activity of the aqueous extract, (2) the plant part that was used for extraction, (3) affordability, (4) availability and common use in the seven countries of the study (Democratic Republic of Congo, Ethiopia, Ghana, Nigeria, Senegal, the Sudan and Uganda) and (5) availability of sufficient and accessible online literature (Figure 1). After selecting the five medicinal plants, available online literature about them was searched for, mainly in PubMed and Google Scholar without timeframe restriction.

**Figure 1:**
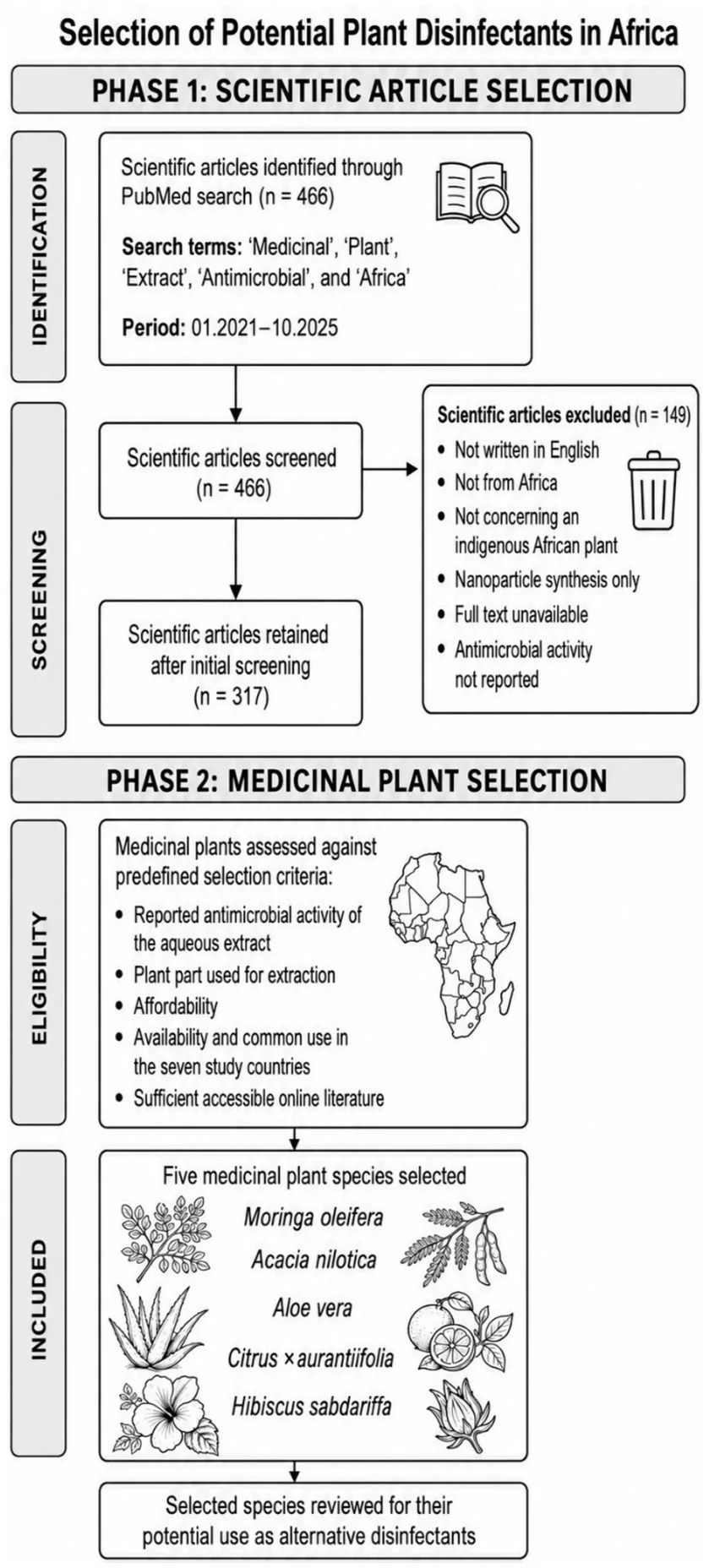
Schematic representation of the methodology for selection of the plants for this review.

## 3 Results

The selected five medicinal plants were *Vachellia nilotica* L. P. J. H. Hurter and Mabb. [synonym *Acacia nilotica* (L.) Willd. ex Delile], *Aloe vera* (L.) Burm.f. (synonym *Aloe barbadensis* Miller), *Citrus ×aurantiifolia* (Christm.) Swingle, *Hibiscus sabdariffa* Linnaeus, and *Moringa oleifera Lam*. The water extracts of these medicinal plants or juices demonstrated antimicrobial activity in various studies (18–22) against many environmental, pathogenic and multidrug-resistant bacteria (MDR), in addition to activities against some fungi, viruses and protozoa. The PubMed search revealed an increasing research interest on these medicinal plants in the last few decades (Supplementary Table S1), with the last 10 years’ literature constituting about 50–80% of the total literature. The following is a brief review on each plant including its habitat and distribution, medicinal uses, chemical compounds and antimicrobial activity, minimum inhibitory concentration and potential use in disinfection.

### 3.1 *Acacia nilotica* (L.) Willd. Ex Delile*/Vachellia nilotica* L. (P. J. H. Hurter and Mabb.)

*Vachellia nilotica* L. (P. J. H. Hurter and Mabb.) or *Acacia nilotica* (L.) Willd. ex Delile is one of the medicinal plants that has been largely used for a long time in ethnomedicine and ethnoveterinary medicine in many countries of Africa and Asia (9, 23, 24), but received neglect in clinical studies (15).

#### 3.1.1 Habitat and distribution

*Acacia* is a pantropical and subtropical genus belonging to family: *Fabaceae*/*Leguminosae*, subfamily: *Mimosoideae* (25) with species abundant throughout Australia, Asia, Africa, and America. *A. nilotica* is widely distributed across tropical and subtropical regions and is traditionally used in many countries where the plant is abundant and easily accessible to local communities.

#### 3.1.2 Medicinal uses

Across traditional medical systems, the gum, stem bark, leaves and fruits of *A. nilotica* are widely used in folk medicine for the treatment of respiratory infections and symptoms such as cough, bronchitis, pneumonia, diarrhea, tuberculosis, dysentery, skin ailments such as leprosy, smallpox, ophthalmia, toothache, parasitic infections, malaria, hemorrhoid and many inflammatory conditions, reflecting its broad ethnopharmacological relevance (26–30). Aqueous extracts of *A. nilotica* have demonstrated anti-inflammatory, antipyretic, analgesic, and antinociceptive activities in animal models, supporting their historical use in managing fever, pain, and inflammation (31). The pods of *A. nilotica* are commonly used in Sudanese ethnomedicine, where they are prepared in various forms to manage infectious conditions, including respiratory diseases such as coronavirus-related symptoms during the pandemic. Its widespread availability and long history of use contribute to its significance as a locally important medicinal plant in many low-resource regions (9).

#### 3.1.3 Chemical compounds and antimicrobial activity

*Acacia nilotica* contains carbohydrates, glycosides, phytosterols, phenolic compounds, saponins, and flavonoids in its hot aqueous leaf extracts, representing the major antimicrobial and immunomodulatory constituents of the plant (29, 32, 33). In addition, tannins, terpenes, and anthraquinones were also detected in ethanolic and/or methanolic (34, 35). Tannins present in the leaves play a major role in ovicidal, larvicidal, and adulticidal activities against parasitic nematodes such as *Haemonchus contortus* (30).

Antimicrobial studies showed that hot aqueous leaf extracts of *A. nilotica* inhibited *Klebsiella pneumoniae*, *Pseudomonas aeruginosa, Escherichia coli, Bacillus cereus, Staphylococcus aureus,* and *Streptococcus uberis*, and suppress growth of *Aspergillus niger* and *Aspergillus fumigatus* (29). Ethanolic leaf extracts further demonstrated antimicrobial and cytotoxic properties, supporting the plant’s traditional therapeutic use (29).

#### 3.1.4 The minimum inhibitory concentration

Only a few studies presented quantitative data on the antimicrobial effect of *A. nilotica* water extracts. One study reported minimum inhibitory concentration (MIC) of 1.6 mg/mL for *E. coli*, and *S. faecalis*, 0.8 mg/mL for *P. aeruginosa*, 0.4 mg/mL for *Proteus mirabilis*, *K. pneumoniae*, *S. aureus* and *Bacillus subtilis*, and 0.1 mg/mL for *Candida albicans* when the plant was extracted in water at 37 °C (18). Another study (36) reported MIC and MBC values of 3.13–12.5 and 25 μg/mL, respectively, for *S. aureus*, *Streptococcus agalactiae* and *E. coli*. Other studies (29, 37, 38) did not provide quantitative MIC measurements and merely relied on inhibition-zone assays. Antiviral assessments similarly presented qualitative inhibitory effects of aqueous extracts from leaves, pods, and bark against Peste des petits ruminant’s virus without determining MIC or half of the maximum inhibitory concentration (IC₅₀) values (39). A concentration-dependent inhibitory value is available from parasitological testing, where an aqueous leaf extract yielded an IC₅₀ of 0.195 mg/mL against *H. contortus* larvae under controlled *in vitro* conditions (30).

#### 3.1.5 Potential use in disinfection

The reported antimicrobial properties of *A. nilotica* enhanced some researchers to explore its potential use for disinfection of water (40–42). Fayyed (40) detected the efficacy of *Acacia* bark for disinfecting polluted water to be used for drinking purposes. He found treatment of 1 L of polluted water with 10 g of *Acacia* bark for 24 h as the optimum conditions for water disinfection. However, Miftahul El Jannah et al. (42) used *A. nilotica* leaves for water treatment. They found a 99.6% reduction in the number of coliform bacteria with 50% concentration of leaves and a reduction in 99.7% with 10% ethanolic extract of the leaves for 24 h treatment. The ethanolic extract of *A. nilotica* was used in a disinfectant formulation and tested against bacteria isolated from the hospital environment. In the suspension test, the *A. nilotica* disinfectant killed all tested bacteria after 10–30 min of exposure (43). In dentistry, the ethanolic extract of *A. nilotica* fruits (pods) was tested for disinfection of addition silicone dental impression material at a concentration of 75 mg/mL for 10 min. A 100% reduction in the number of both Gram-positive and Gram-negative bacteria was obtained (44). Jagyasi et al. (45) concentrated the water extract of *A. nilotica* to 1 g/mL by heating at 300 °C and tested it for disinfecting the gutta-percha cones. They found no significant difference between the bark extract and 2% chlorhexidine in decontaminating the cones. When used as mouth rinse for 30 days, the *A. nilotica* gave similar results of those of 0.02% chlorhexidine in reducing mutans streptococci in high-caries risk volunteers (46).

### 3.2 *Aloe vera* (L.) Burm.f

*Aloe vera* (L.) Burm.f. (synonym *Aloe barbadensis* Mill.) is one of the few medicinal plants that have been used for millennia in ethnomedicine and has found acceptance by modern Western societies for use as a home medication (47, 48). In recent years, *Aloe vera* has been one of the most widely studied medicinal plants. From 2001 to 2020 Adetunji et al. (49) identified about 8,200 scientific publications that have been published on the genus *Aloe*, with 5% annual growth rate, the majority of which were on *A. vera*. Updates of the increasing interest of the scientific community on this plant can be seen in Supplementary Table S1.

#### 3.2.1 Habitat and distribution

*Aloe vera* belongs to the genus *Aloe*, the members of which are native to the tropical and southern Africa, the Arabian Peninsula, Madagascar, and associated Indian Ocean islands (50). Generally, it grows in dry regions of Africa, Asia and the Americas (51).

#### 3.2.2 Medicinal uses

*Aloe vera* is used in many parts of the world for the treatment of various conditions including intestinal problems such as colic, constipation, helminths infestation, in addition to its famous uses for skin problems (47, 52). Furthermore, several investigations have shown its beneficial effects on the management of metabolic syndrome as anti-diabetic, anti-hypertensive, lipid-lowering, anti-obesity and as well as having cardioprotective impacts (53). Therefore, it is included in many pharmaceutical and skincare formulations, as well as in some food such as beverages (47).

#### 3.2.3 Chemical compounds and antimicrobial activity

Antimicrobial activity of *Aloe vera* was documented early (54) and have been shown later in several publications around the world (54–65). Many bioactive compounds have been identified in *A. vera*, to which the antimicrobial activities are referred to. As have been listed by Catalano and others (66), they include anthraquinones and anthrones (Aloe-emodin, emodin, aloin A [barbaloin)] aloin B [isobarbaloin], rhein [cassic acidm monorhein, rheic acid, rhubarb yellow), chrysophanol [chrysophanic acid], aloetic acid, aloesaponarin I, aloesaponarin II, deoxyerythrolactin, laccaic acid D-methylester, helminthosporin, aloesin [aloeresin B]; glycosides: glycosylated chromones (aloeresin A, aloeresin D, aloe-aloeresin D, Rebaichromone], glycosylated pyran-2-ones (Aloenin or Aloenin AAloen, Aloenin B), coumarins and isocoumarins (feralolide, esculetin), polisaccharides (acemannan). Aloe-emodin has antiviral (67–69), antibacterial (70), antibiofilm formation (71), and antiparasitic effects (66). Emodin has antibacterial activity including MDR *Mycobacterium tuberculosis* and some other bacteria (72–74), antiviral activity (75, 76) and antifungal activity (77). These activities have been shown for the gel and/or the leaves in different extracts (aqueous, ethanolic, methanolic, etc.) of the plant.

#### 3.2.4 The minimum inhibitory concentration

Antimycobacterial activity was investigated at concentrations of 0.25–8 mg/mL, with 95% bactericidal effect for 1 week (78). *A. vera* gel showed significant (*p* < 0.001) growth inhibition of *Shigella flexneri* at gel concentrations greater than 112 mg/mL when compared with the control (79). The aqueous extract of *A. vera* showed antibacterial activity against *S. aureus*, *Shigella* spp., and *E. coli*, with inhibition zones of 1.10 ± 0.10, 0.47 ± 0.25, and 0.40 ± 0.10 mm, respectively (80). In another study (22), the MICs against different Gram-negative bacteria including MDR strains varied from 0.625 to more than 20 mg/mL and the minimum bactericidal concentration (MBC) from 1.25 to more than 20 mg/mL. However, the MIC values of specific *A. vera*- derived phytochemicals (Aloin, Aloe-emodin, Saponins, Salicylic acid, Acemannan, Lupeol and Cinnamic acid) against MDR- *P. aeruginosa* ranged from 125 to 500 μg/mL (81). Also, for emodin an MIC of 16 μg/mL was reported against *Streptococcus suis* (82). For fungi, an MIC of 1:500 was reported for aqueous extract against different clinical isolates of *Aspergillus* (*A. fumigatus, A. glaucus,* and *A. tereus*) and *Candida* (*C. albicans, C. glabrata,* and *C. tropicalis*) (77).

#### 3.2.5 Potential use in disinfection

Evaluation of *Aloe vera* alone for use in disinfection has been addressed by few studies, mostly testing disinfectant formulations containing *Aloe vera* gel among other components in hand sanitizers (83), dentistry and mouth hygiene (84–87). The results of these studies showed little or no efficacy against the bacteria that were tested. Other studies evaluated mouth wash formulations containing *Aloe vera* by their activity in reducing the plaque formation or gingivitis incidence (88), without direct testing of the antimicrobial activity. However, one study tested the efficacy of the gel on the disinfection of water line of a dental unit and found that *Aloe vera* was the most effective in water disinfection compared with 5% sodium hypochlorite and 10% hydrogen peroxide (85).

### 3.3 *Hibiscus sabdariffa* L

*Hibiscus sabdariffa* L. is one of the well-known food and medicinal plants in many parts of the tropical countries in Africa and Asia, especially for its delicious drink that are made from its calyx, which is consumed hot or cold. In the Sudan, it is known as Karkadeh (89) and has different local names in other countries; however, the English name is known as Roselle.

#### 3.3.1 Habitat and distribution

*Hibiscus sabdariffa* is native to the tropics (90), and now is widely cultivated throughout tropical and subtropical areas in Africa, Asia, and the Americas.

#### 3.3.2 Medicinal uses

*Hibiscus sabdariffa* in ethnomedicine is used to treat a number of ailments across tropical countries. Ross (90) summarized some therapeutic uses of the plant parts (root, leaves, flowers, seed) by people in many tropical countries in Africa, Asia and America (Supplementary Table S2): as agent for lowering blood pressure, diuretic, choleretic, cholagogue, laxative, diaphoretic and for cough (90). In Uganda, it is used for the treatment of anaemia (91). In Senegal and the Sudan, it is used for the treatment of cold and fever (92–94), and malaria with other herbs (95), namely, *Tamarindus indica*.

#### 3.3.3 Chemical compounds and antimicrobial activity

The chemical compounds of *Hibiscus sabdariffa* are not fully identified. Most studies provided only broad lines, with differences in the extraction methods and the plant part that has been extracted. Anthocyanins, phenolics, flavonoids, saponins, tannins, triterpenoids, glycosides and fatty acids in the calyces (96–98), with phenolics being the major concern in many studies testing the antimicrobial activity of the plant extracts. Suniarti et al. (98) identified many chemical compounds in the ethanolic extract of calyces of this plant to which they related antimicrobial (antibacterial, antifungal or antiviral) activities, as they discussed. These included, among others, hexadecanoic acid, organosulfur, alkyl halides, benzothiazoles and furans.

Aqueous calyx extracts exhibited *in vitro* and *in vivo* antibacterial activity against MDR enterohemorrhagic *E. coli* and *Salmonella Typhimurium*. In infected CD-1 mice, oral administration of aqueous extract eliminated detectable enterohaemorrhagic *E. coli* (EHEC) and *Salmonella Typhimurium* from feces within 2–3 days, whereas untreated animals continued shedding pathogens and some succumbed to infection (99). Some studies also demonstrated activity against hospital isolates of MDR *Acinetobacter baumannii* using calyx aqueous extracts (100) and against *Salmonella Typhi* in addition to synergistic interactions between aqueous extract and selected antibiotics *in vitro* (51, 101).

Although many studies showed antifungal activities against *C. albicans* strains for aqueous (102), 80% aqueous ethanolic (103) or methanolic extracts (104) of *Hibiscus sabdariffa*, one study (105) showed no activity against a *C. albicans* strain for both aqueous and ethanolic extracts, probably due to strain differences. Moreover, antiviral activities of *Hibiscus sabdariffa* extracts have been shown against hepatitis A virus, feline calicivirus, murine norovirus (106) and Aichi virus (107).

#### 3.3.4 The minimum inhibitory concentration

Aqueous extracts of *Hibiscus sabdariffa* showed measurable antibacterial potency, with MIC and MBC values of 20 and 50 mg/mL, respectively, against MDR EHEC *E. coli* and *Salmonella Typhimurium* in a murine infection model (99). Similarly, aqueous extracts inhibit enteropathogenic *E. coli* (EPEC) by preventing pedestal formation and inducing bactericidal effects, including filamentation, with a reported MIC of 6.5 mg/mL (20).

The *Hibiscus sabdariffa* aqueous extract at a concentration of 40 mg/mL reduced the titers of three viruses from 5 log PFU/ml to undetectable levels after 15 min for feline calicivirus (FCV-F9), by 24 h for murine norovirus (MNV-1) and after 24 h for hepatitis A virus (106). Aichi virus was reduced to undetectable levels after 24-h exposure to aqueous extracts at concentration of 40 mg/mL (107).

#### 3.3.5 Potential use in disinfection

There is no available literature on the use of *Hibiscus sabdariffa* extracts as disinfectant or antiseptic. However, the documented activity of aqueous *Hibiscus sabdariffa* extract against MDR bacteria and some viruses, in addition to its activity against many fungi and yeasts suggests its potential use as a locally accessible antimicrobial agent in settings with limited availability of chemical disinfectants.

### 3.4 *Citrus ×aurantiifolia* (Christm.) swingle

*Citrus ×aurantiifolia* (Christm.) Swingle (or *Citrus aurantifolia*) belongs to the Family *Rutaceae* (108) and is a known fruit for its delicious juice.

#### 3.4.1 Habitat and distribution

Like other *Citrus* species, *C. aurantiifolia* is a perennial tree originating from the wet tropical regions of Southeast Asia. It is now widely cultivated across the tropics and subtropics, where climatic conditions support large-scale commercial production. Citrus cultivation extends broadly between 40°N and 40°S, reaching elevations up to 1800 m in tropical zones and 750 m in subtropical regions (109).

#### 3.4.2 Medicinal uses

*Citrus ×aurantiifolia* (lime) is widely used in ethnomedicine for the treatment of infectious and inflammatory conditions. Ethnopharmacological surveys describe its use for various ailments and infections, reflecting a long tradition of employing lime preparations in folk medicine (110). In Indonesia, the fruit juice is used in treatment of Flu-cough, sore throat (111), hemoptysis associated cough of tuberculosis (TB) (112), in Nigeria, lime juice is used to suppress stomach pain, mixed with sugar and palm oil or with honey to relief cough and to prevent pimple when used as facial scrub (113), in addition to its use in therapies for malaria and constipation (114). In the Sudan, the fruit juice in added to tea for relieving common cold, to coffee for diarrhea and is applied on fresh wounds to prevent infection.

#### 3.4.3 Chemical compounds and antimicrobial activity

Phytochemical analyses of *C. aurantiifolia* demonstrate a complex profile of bioactive secondary metabolites. Juice concentrates and peel and leaf extracts contain alkaloids, flavonoids, steroids, saponins, phenols; glycosides in juice and peel; tannins and terpenoids juice and leaf extract (115). Phenolic compounds and tannins were reported in black dry lime fruit extracts (116). Reviews of lime peel essential oil highlight limonene as a major antibacterial component contributing to the antimicrobial activity of peel-derived preparations (117).

Lime juice, peel, leaf, seed and burnt rind (black dry lime) extracts inhibit a wide range of Gram-positive and Gram-negative bacteria, anaerobes and yeasts. *In vitro* studies reported activity against *S. aureus*, *Streptococcus pyogenes*, *Streptococcus mutans*, *Enterococcus faecalis* (reported as *Streptococcus faecalis*), *Bacillus* spp., *Actinobacillus* spp., *Corynebacterium* spp., *Propionibacterium acnes*, *Listeria monocytogenes*, *E. coli*, *Salmonella Typhi, Salmonella Paratyphi* A and B*, K. pneumoniae, Klebsiella oxytoca, P. aeruginosa, Citrobacter* spp.*, Serratia* spp., *Hemophilus influenza*, as well as *C. albicans* and, for oil extracts, *A. niger* (113, 118–126). Lime juice and extracts also showed activity against methicillin-resistant *Staphylococcus aureus* (MRSA), *P. aeruginosa* and environmental pathogens relevant for surface contamination (21). Black dry lime extracts inhibited growth and biofilm formation of *E. coli* reference strains (116).

#### 3.4.4 The minimum inhibitory concentration

Quantitative susceptibility data for *C. aurantiifolia* preparations showed measurable MIC and MBC ranges against clinically relevant bacteria and fungi. Using lime juice, burnt rind preparations and steam-distilled oil, *in vitro* assays reported that anaerobes and Gram-positive bacteria (including *S. aureus* and *E. faecalis*) were inhibited at MIC values between 32 and 128 mg/mL, whereas Gram-negative bacteria such as *E. coli*, *K. pneumoniae, P. aeruginosa, Salmonella Paratyphi, S. flexneri*, *Citrobacter* spp. and *Serratia* spp. showed MIC values between 64 and 512 mg/mL; *C. albicans* exhibited MIC values of 256–512 mg/mL, and only the oil extract was active against *A. niger*. Corresponding MBC values for these organisms ranged from 32 to 512 mg/mL, depending on the species and solvent used (113).

For citrus juice concentrates, including lime, MIC and MBC values against bacterial strains ranged from 12.5 to 200 μg/mL, while minimum fungicidal concentrations against tested fungi were reported between 50 and 200 μg/mL, with lime concentrates generally showing lower MIC and MBC values than orange or tangerine (115). However, aqueous extracts of black dry lime displayed relatively high MIC and MBC values (~6,800–10,200 μg/mL) different against *E. coli* ATCC 25922 and ATCC 35218 (116).

#### 3.4.5 Potential use in disinfection

Several studies specifically address the potential of *C. aurantiifolia* preparations as natural disinfectants. Lime juice has been evaluated for environmental hygiene: in a kinetic growth model, key lime juice rapidly eliminated *P. aeruginosa* and MRSA *in vitro*, and a mixture of commercial disinfectant detergent with lime juice effectively reduced microbial contamination on fomite surfaces in laboratory settings. However, these findings suggest that lime juice can enhance or complement conventional detergents for surface disinfection in healthcare and laboratory environments (21).

### 3.5 *Moringa oleifera* lam

*Moringa oleifera* Lam. (the drumstick plant) is widely recognized for its therapeutic uses in ethnomedicine for treatment various infections and ailments (127). It represents one of the widely researched medicinal plants worldwide. The research interests on this plant increased dramatically within the last 10 years, with the articles published in this year accounting to almost 15% of the total articles on it in PubMed since 1946 (Supplementary Table S1).

#### 3.5.1 Habitat and distribution

*Moringa oleifera* is native to the Indian subcontinent but now it is widely cultivated throughout tropical and subtropical regions, including Africa, South America, Asia, and the Middle East (128, 129). The species can grow under harsh conditions and is cultivated in arid regions, where it has been introduced for sustainable rural development (130). Because of its adaptability and broad distribution, *M. oleifera* has become an important multipurpose crop in many low- and middle-income countries (128, 129, 131).

#### 3.5.2 Medicinal uses

The plant is applied in local health practices due to its antimicrobial, nutritional, and coagulant properties, supporting its use in household and community medicine (132, 133). Its therapeutic uses in ethnomedicine include heart disease, anaemia, arthritis, erectile, dysfunction in Ghana (134), hypertension, fever, and as antidote for detoxification of poisons in Thailand (135).

#### 3.5.3 Chemical compounds and antimicrobial activity

Antimicrobial properties of *M. oleifera* were investigated as early as 1946 (136) during the eras of discoveries of antibiotics and the search for antibiotics from natural products. Later, extensive *in vitro* studies confirmed that *M. oleifera* extracts exhibit strong antimicrobial activity against a wide spectrum of environmental and pathogenic bacteria and fungi, such as *S. aureus*, MRSA, *S. faecalis*, *Clostridium perfringens*, *L. monocytogenes*, *Microcystis aeruginosa*, *Bacillus anthracis*, *Salmonella enteritidis*, *E. coli*, *Proteus vulgaris*, *P. aeruginosa*, *Candida* spp., and *Fusarium* spp. (127, 128, 130–133, 137, 138).

*Moringa oleifera* leaves and seeds contain many bioactive compounds to which the antimicrobial activities have been linked to. *M. oleifera* leaves contain phenolic acids such as gallic acid, ferulic acid, and caffeoylquinic acid, together with flavonoids including quercetin, luteolin-7-glycoside, kaempferol, rutoside, and myricetin (128–130, 137, 139, 140). The seeds contain moringin – a benzyl isothiocyanate derivative (141). *M. oleifera* leaf polysaccharides extracted by hot water showed antibacterial activities against *S. aureus*, *B. subtilis*, *E. coli* and *P. aeruginosa*, in addition to antibiofilm activities (142). Also, moringin showed antimicrobial activity against many bacterial strains with antimicrobial resistance genes such as *L. monocytogenes* and *S. aureus* and also suppressed biofilm formation (143).

#### 3.5.4 The minimum inhibitory concentration

Aqueous leaf extract of *M. oleifera* exhibited bactericidal activity at very low concentrations; it showed an MIC of 0.078 mg/mL for *P. aeruginosa*, 0.156 mg/mL for *E. faecium*, and 5 mg/mL for both *E. coli* and *S. aureus* (144), but showed a higher MIC (12.5 mg/mL) for MRSA with inhibition zones increasing with increased concentration (145). The MBC was reported to be 1.25 mg/mL for *P. aeruginosa*, 2.5 mg/mL for *E. faecium* and 10 mg/mL for *E. coli* and *S. aureus* (144).

#### 3.5.5 Potential use in disinfection

*Moringa oleifera* has been widely investigated as a natural coagulant and antimicrobial agent for water and wastewater treatment (19, 132, 133, 146–150). Seed powder has been tested as an antibacterial agent and coagulant in river and stream water, achieving up to 99.4% reduction of *Salmonella* spp., 78.8% for *E. coli*, and 57.3% for *S. aureus* at a dose of 0.017 g/mL, alongside improvements in water hardness and ionic composition (19).

A systematic review of water-treatment applications indicated that antimicrobial studies using leaf or seed extracts obtained with distilled water or 95% ethanol, frequently reported about 30 mg/mL as particularly effective against *E. coli* and potentially *Vibrio cholerae* (132). For greywater in Ghana, *M. oleifera* seed powder combined with sand filtration improved turbidity, solids, nutrients, and microbial quality of bath wastewater. In that study, turbidity and Total Suspended Solids were reduced by about 98–99%, and *E. coli* and total coliform counts decreased by more than 99.99%, indicating effective microbial and particulate removal (151). Therefore, *M. oleifera* seed extract was suggested a cost-effective option for water clarification and primary disinfection, especially in rural communities without access to conventional water treatment (132, 146).

## 4 Notes on the plant extracts

The criteria for selecting the plants for this review were set to support the aim of their use in sub-Saharan African resource-limited settings. The water extraction criterion avoids other extracts that require expensive materials and special equipment or complicated techniques that suit industrial communities. The plant part used for extraction was an important consideration for perennial plants; using the root or bark endangers the plant’s existence and results in negative environmental impacts. Local availability can reduce vulnerability to supply-chain disruption, while affordability excludes prohibitively expensive plants.

It should be noted that the available literature evaluating the antimicrobial activity of medicinal plant extracts provides assay results measuring the MIC, MBC or inhibition zone. When these assays prove an *in vitro* activity of a medicinal plant extract or product against the tested organism(s), they indicate a possible *in vivo* therapeutic activity and potential disinfectant activity as well. Therefore, the reported antimicrobial properties of the aqueous extracts of different parts of the medicinal plants *Acacia nilotica, Aloe vera*, *Citrus ×aurantiifolia*, *Hibiscus sabdariffa* and *Moringa oleifera* combined with the simple preparation methods meet the criteria set for their selection in this review and support their suitability for use in disinfection in resource-limited settings in the countries of the study.

Furthermore, the reported antimicrobial spectrum of extracts of these medicinal plants spans many medical and veterinary important pathogens, such as *E. coli* and ESKAPE bacteria, including many MDR and biofilm-forming strains (12). This broad spectrum of activity positions them as potential alternatives for disinfection of veterinary areas.

While recent studies support the antimicrobial potential of medicinal plant-derived compounds, they highlight the need for *in vitro* and *in vivo* evaluation of their activity and toxicity (12, 152).

However, the following practical considerations apply to individual plants:

*Acacia nilotica*: The water extraction should be performed at temperature below 55 °C because the antimicrobial activity of water extract decreases with the increase of water temperature, as it was clear from the report of Abd El Nabi et al. (18).

*Aloe vera*: The antimicrobial activity of *Aloe* gel decreases with time, therefore it should be used in the fresh form immediately after maceration from the leaves, or freezing it after maceration until use (54).

*Citrus ×aurantiifolia*: Lime juice and extracts of peel and leaf possess antimicrobial activity.

*Moringa oleifera*: Both seed and leaf extracts possess antimicrobial activity. The seeds should be ground before extraction.

## 5 Limitations for using the plant extract in disinfection

Despite the long use of medicinal plants in ethnomedicine and their beneficial effects that have been proven by many studies, limitations associated with their therapeutic use, e.g., the exact therapeutic dose and concentration, also apply to their use for disinfection. Furthermore, the methods of evaluating antimicrobial effects that justify their use in disinfection are inconsistent among the different studies, in addition to the limited number of studies evaluating their use as disinfectants.

Testing the efficacy of these extracts for disinfection in the veterinary field is performed under controlled laboratory conditions following standard protocols, such as the European Standards for quantitative testing the bactericidal activity of disinfectants and antiseptics in the veterinary area in suspension or on surfaces (e.g., EN 1656, EN 14349/16437). However, the current literature about these medicinal plants is deficient in this respect.

The costs might be one of the limitations when medicinal plant extracts are to be used on a large scale for disinfection, as disinfection normally requires large volumes of disinfectants. However, the costs of medicinal plants vary from country to country and from one area to another within the country and from season to season. In areas where a plant is naturally growing, its costs for use in disinfection would be minimal. Furthermore, the focus of this review is on the water extracts, the costs of which are very low. The total costs of large-scale production, from harvesting through extraction to the finished aqueous extract, have not been quantified for these medicinal plants extracts in the available literature, and dedicated cost-effectiveness studies are required before large-scale adoption in veterinary settings can be recommended.

## 6 Suggested uses of the plant extract in disinfection

Taking into consideration the limitations addressed above in section 5, the uses suggested below for the plant extracts reviewed here (illustrated in Figures 2, 3) might help in guiding future evaluation studies:

1. *Acacia nilotica* pod extracts can be used in foot disinfection dips at entrances of farms and stalls, disinfection of animal houses and premises and disinfection of surfaces; the bark extract can be used for water treatment.
2. *Aloe vera* gel can be used for hand disinfection, e.g., before and after milking, teat disinfection and surface disinfection.
3. *Citrus ×aurantiifolia* juice and peel extracts can be used for surfaces disinfection, hand hygiene and udder hygiene; the leaf extract can be used for water disinfection.
4. *Hibiscus sabdariffa calyx extract can be used for surface disinfection, water disinfection and in teat dips.*
5. *Moringa oleifera* seed extract is suitable for disinfection in water-treatment systems, the leaf extract for surface disinfection and hand hygiene.

**Figure 2:**
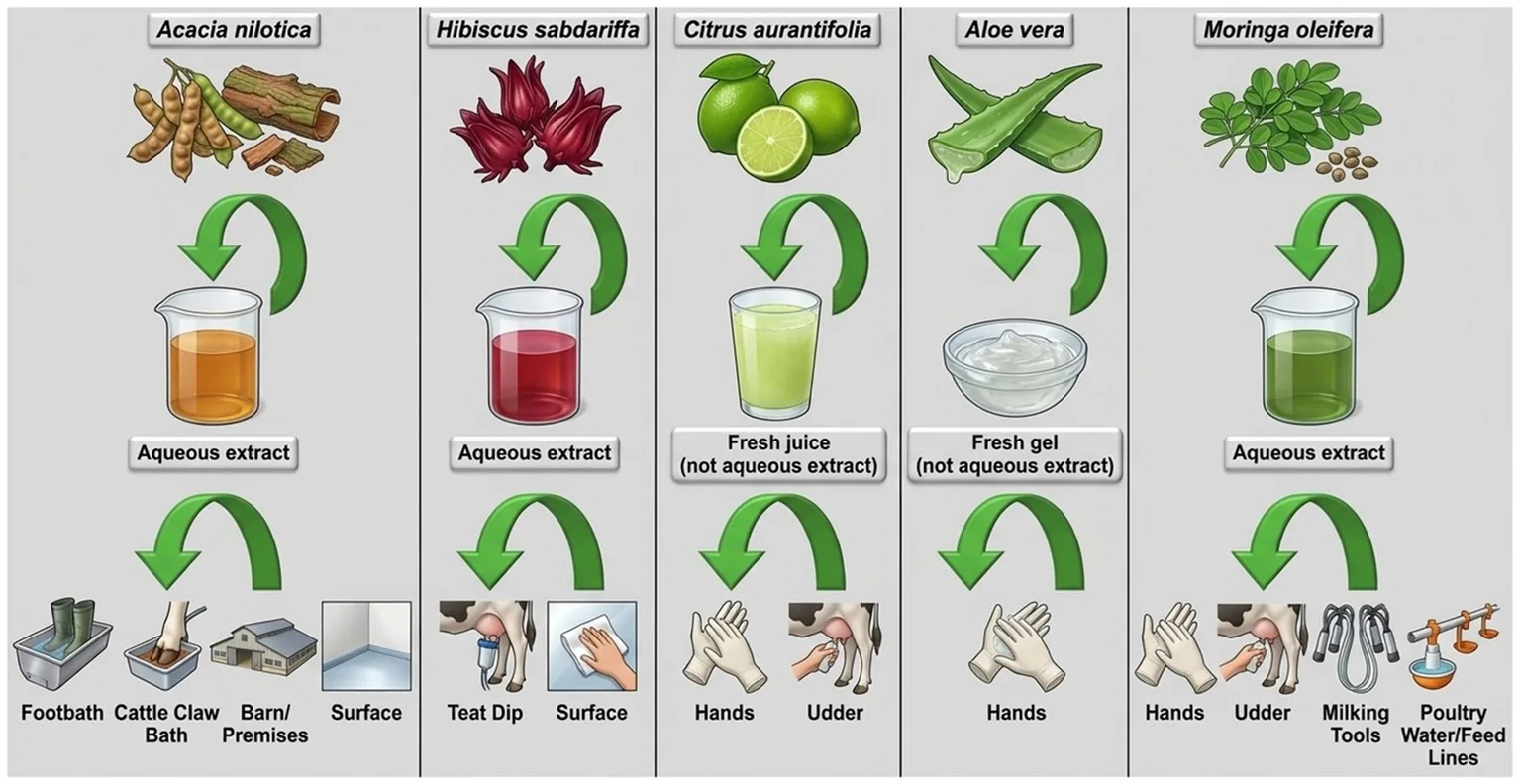
Suggested applications of the plant-based disinfectants in dairy cattle setting. All preparations represent aqueous extracts, except *Aloe vera* (fresh gel) and *Citrus ×aurantiifolia* (fresh juice).

**Figure 3:**
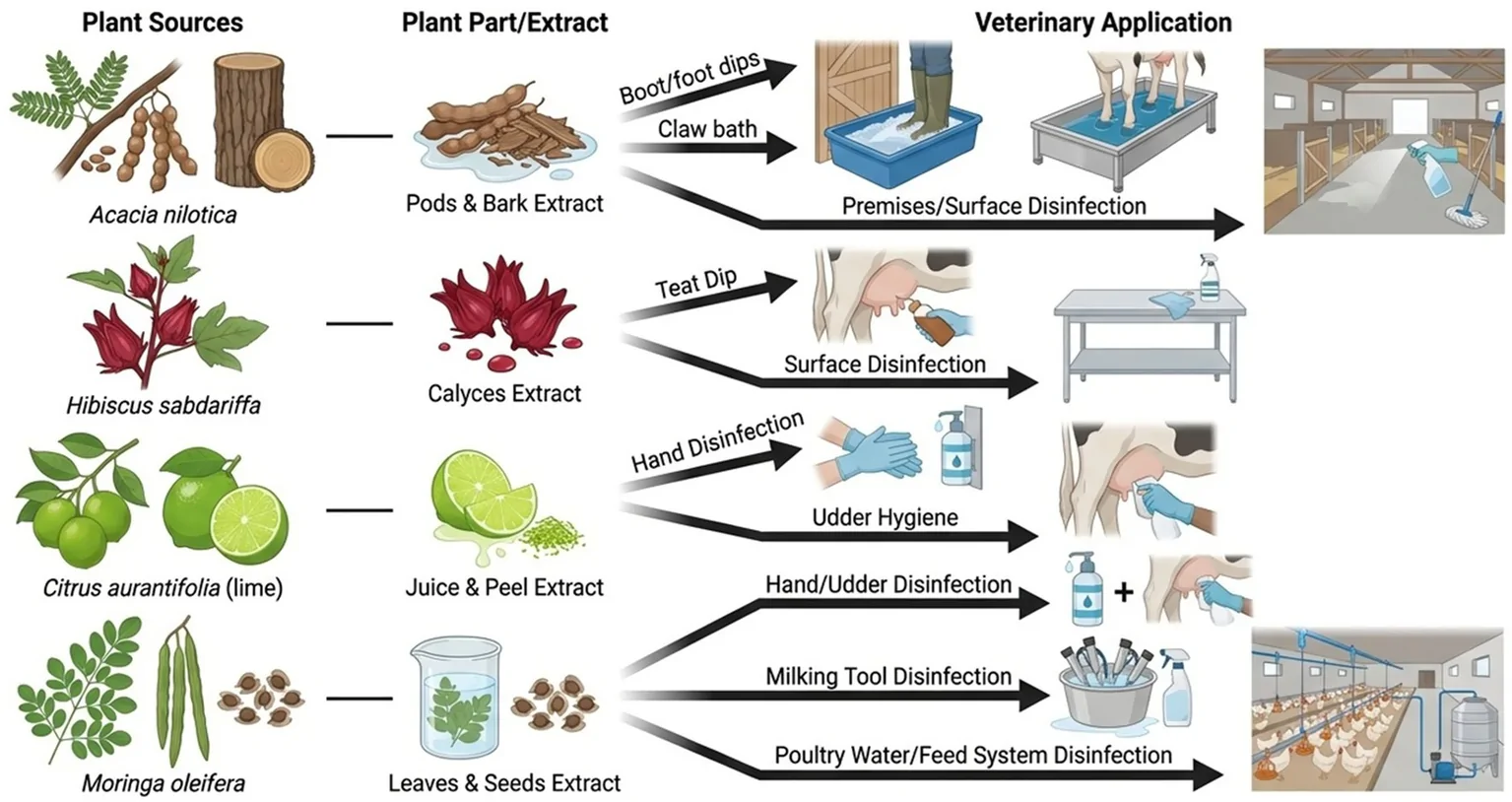
A wider scheme for the application of the plant-based disinfectants in veterinary settings.

It is noteworthy that in ethnomedicine in the Sudan, the pods of *Acacia nilotica* are burned in charcoal, and their smoke is inhaled for the treatment of respiratory tract infections, such as sore throat or common cold. This practice was widely used as effective traditional treatment of coronavirus infection. However, further experimental and controlled studies are required to assess the effectiveness and safety of *Acacia nilotica* pod fumigation for disinfection purposes in the veterinary settings, such as animal housing, poultry incubators and hatcheries. Similar studies are also required for evaluating the use of *Aloe vera*, *Citrus ×aurantiifolia*, *Hibiscus sabdariffa* and *Moringa oleifera*.

## 7 Conclusion

Little information on the MIC of extracts of these medicinal plants is obtainable from the present literature. Nevertheless, the reported antimicrobial activity of their water extracts, together with their accessibility and low cost, suggest that they are worth evaluating as disinfectant options in veterinary settings. Their long use as food and in traditional medicine suggests a favorable safety profile, although safety at the concentrations required for disinfection has not been assessed. These medicinal plants are widely distributed, locally known, and can be processed with simple techniques such as drying, grinding, and decoction without sophisticated equipment—techniques that are deeply embedded in community practice. Beyond the suggested use in veterinary settings, they may also merit exploration as a plant-based resource for community-level disinfection and hygiene applications, for contamination control in environments where foodborne and waterborne transmission scenarios are possible and for water-treatment solutions.

Besides their safety, availability, sustainability and affordability, these medicinal plants represent promising candidates for animal farmers in resource-limited areas, especially during times of disruption of the global supply chains, or when chemical disinfectants are scarce or expensive. Special official and research efforts should be directed toward standardizing the extraction protocols, specifying the effective concentrations, and evaluating these plant extracts using standard disinfectants-testing protocols. Therefore, the suggested uses of the plant extracts mentioned in this review are only guiding notes for further research and for rigorous evaluation by peers and regulatory bodies.
