# Supplementary material for: Plant extracts as alternatives for chemical disinfectants in veterinary settings in seven sub-Saharan African countries
Source: Front Vet Sci. 2026 Sep 30;13:1895590. doi: 10.3389/fvets.2026.1895590 (PMC13663173; doi:10.3389/fvets.2026.1895590)
Supplement: Supplementary file 1 [file Supplementary_file_1.docx]

Elhadi et al. (*Front. Vet. Sci.* 13:1895590; doi: 10.3389/fvets.2026.1895590)

**Supplementary Tables**

# **Table S1: Online available literature on the five plants of the review based on PubMed search results on 22/12/2025, 05:00 pm CET**

| **Period** | **Plant** | **Years** | | | |
| --- | --- | --- | --- | --- | --- |
|  |  | **Whole** | **10** | **5** | **1** |
| 1952-2025 | *Hibiscus sabdariffa* | 824 | 541 (66%) | 310 (38%) | 57 (7%) |
| 1946-2026 | *Moringa oleifera* | 2540 | 2066 (81%) | 1389 (55%) | 363 (14%) |
| 1868-2026 | *Aloe vera* | 5315 | 2746 (52%) | 1697 (32%) | 375 (7%) |
| 1982-2026 | *Acacia/Vachellia nilotica* | 375 | 220 (59%) | 143 (38%) | 26 (7%) |
| 1946-2025 | *Citrus ×aurantiifolia* | 462 | 243 (53%) | 136 (29%) | 32 (7%) |

# **Table S2: Traditional medicinal uses of *Hibiscus sabdariffa* according to Ross (2003) ***

| **Africa** | Hot water extract of seeds is taken as a diuretic and tonic. Seed oil is used externally to heal sores on camels. |
| --- | --- |
| **Brazil** | Hot water extract of root is taken orally as a stomachic and externally as an emollient. |
| **Cameroon** | Hot water extract of dried leaves is taken orally as an anthelmintic. |
| **Congo** | Hot water extract of leaves is taken orally to expedite delivery. |
| **East Africa** | Hot water extract of leaves is taken orally to relieve coughs. Unripe fruit juice is taken orally with salt, pepper, asafetida and molasses as a remedy for biliousness. Hot water extract of leaves is used as a flavouring agent, diuretic, choleretic, febrifuge and hypotensive, to decrease viscosity of blood and to stimulate intestinal peristalsis. Externally, the extract is used for sores and wounds. |
| **Egypt** | Decoction of hot water extract of the calyx is taken with sugar 3 times daily for high blood pressure. Hot water extract of the entire plant is taken orally for heart and nerve diseases, as a laxative, to reduce weight, as a diuretic, to activate and neutralize hepatic secretion, to activate gastric secretion, as a digestive, for arteriosclerosis, as a diaphoretic, to give a euphoric impression and as an intestinal antiseptic. Leaf essential oil is taken orally to treat cancer. |
| **Guatemala** | Hot water extract of dried calyx is taken orally as a diuretic and for renal inflammation. |
| **Guinea-Bissau** | Seeds are taken orally by males as an aphrodisiac. |

*Ross IA. "Hibiscus Sabdariffa". In: Medicinal Plants of the World, 2nd Edn. Totowa, NJ: Humana Press (2003)
